# Clinical Inquiries Regarding Ebola Virus Disease Received by CDC — United States, July 9–November 15, 2014

**Published:** 2014-12-12

**Authors:** Mateusz P. Karwowski, Elissa Meites, Kathleen E. Fullerton, Ute Ströher, Luis Lowe, Mark Rayfield, Dianna M. Blau, Barbara Knust, Jacqueline Gindler, Chris Van Beneden, Stephanie R. Bialek, Paul Mead, Alexandra M. Oster

**Affiliations:** 1 Epidemic Intelligence Service, CDC; 2 Epidemiology/Laboratory Task Force, 2014 Ebola Response Team, CDC; 3 Division of Environmental Hazards and Health Effects, National Center for Environmental Health, CDC; 4 Division of STD Prevention, National Center for HIV/AIDS, Viral Hepatitis, STD, and TB Prevention, CDC; 5 Division of Health Informatics and Surveillance, Center for Surveillance, Epidemiology, and Laboratory Services, CDC; 6 Division of High-Consequence Pathogens and Pathology, National Center for Emerging and Zoonotic Infectious Diseases, CDC; 7 Division of Preparedness and Emerging Infections, National Center for Emerging and Zoonotic Infectious Diseases, CDC; 8 Division of Global Disease Detection and Emergency Response, Center for Global Health, CDC; 9 Global Immunization Division, Center for Global Health, CDC; 9 Division of HIV/AIDS Prevention, National Center for HIV/AIDS, Viral Hepatitis, STD, and TB Prevention, CDC; 10 Division of Bacterial Diseases, National Center for Immunization and Respiratory Diseases, CDC; 11 Division of Viral Diseases, National Center for Immunization and Respiratory Diseases, CDC; 12 Division of Vector-Borne Diseases, National Center for Emerging and Zoonotic Infectious Diseases, CDC

Since early 2014, there have been more than 6,000 reported deaths from Ebola virus disease (Ebola), mostly in Guinea, Liberia, and Sierra Leone (1). On July 9, 2014, CDC activated its Emergency Operations Center for the Ebola outbreak response and formalized the consultation service it had been providing to assist state and local public health officials and health care providers evaluate persons in the United States thought to be at risk for Ebola. During July 9–November 15, CDC responded to clinical inquiries from public health officials and health care providers from 49 states and the District of Columbia regarding 650 persons thought to be at risk. Among these, 118 (18%) had initial signs or symptoms consistent with Ebola and epidemiologic risk factors placing them at risk for infection, thereby meeting the definition of persons under investigation (PUIs). Testing was not always performed for PUIs because alternative diagnoses were made or symptoms resolved. In total, 61 (9%) persons were tested for Ebola virus, and four, all of whom met PUI criteria, had laboratory-confirmed Ebola. Overall, 490 (75%) inquiries concerned persons who had neither traveled to an Ebola-affected country nor had contact with an Ebola patient. Appropriate medical evaluation and treatment for other conditions were noted in some instances to have been delayed while a person was undergoing evaluation for Ebola. Evaluating and managing persons who might have Ebola is one component of the overall approach to domestic surveillance, the goal of which is to rapidly identify and isolate Ebola patients so that they receive appropriate medical care and secondary transmission is prevented. Health care providers should remain vigilant and consult their local and state health departments and CDC when assessing ill travelers from Ebola-affected countries. Most of these persons do not have Ebola; prompt diagnostic assessments, laboratory testing, and provision of appropriate care for other conditions are essential for appropriate patient care and reflect hospital preparedness.

As part of CDC’s Emergency Operations Center activation, CDC staff assist state and local health departments to evaluate PUIs for Ebola. PUIs are defined as persons who, based on initial screening and clinical assessment, have 1) signs or symptoms consistent with Ebola* and 2) an epidemiologic risk factor† within the 21 days before symptom onset (2,3). CDC recommends testing for Ebola virus when persons are confirmed to have compatible clinical presentations and epidemiologic risk factors. For clinical inquiries that resulted in Ebola virus testing, tests were conducted in local or state public health laboratories, most of which are part of the CDC Laboratory Response Network, or in the CDC laboratory (4).

For this report, CDC reviewed inquiries concerning potential PUIs received by CDC during July 9–November 15, 2014, from U.S. health departments or health care providers. Information was compiled from call logs to assess source of inquiry, the person’s travel history or other risk factors for Ebola, clinical presentation, and subsequent Ebola test results.

During July 9–November 15, 2014, CDC responded to clinical inquiries regarding 650 persons from 49 states and the District of Columbia (Figure 1); 142 (22%) originated in health departments, and 508 (78%) were originated by clinicians with subsequent notification and engagement of the health department of jurisdiction. Among persons for whom demographic information was provided, 49% were female, median age was 34 years (range = 9 months–90 years), and 16% were aged <18 years (information on sex and age were available for 82% and 66%, respectively). Overall, 138 (21%) persons had traveled to an Ebola-affected country, 22 (3%) had contact with an Ebola patient or patient’s body fluids in the United States, and 490 (75%) had neither risk factor. Among the 160 persons who had an epidemiologic risk factor, 118 (74%) had at least one sign or symptom consistent with Ebola and therefore met PUI criteria. Inquiries concerning PUIs originated in 34 states and the District of Columbia. Inquiries averaged 10 per week (range = 1–25) until September 30, 2014, when CDC confirmed the first Ebola case diagnosed in the United States (7); after this, the number of weekly clinical inquiries increased, peaking at 227 in mid-October. Most of the increase in inquiries was related to persons with no risk factors for Ebola (Figure 2).

A total of 61 (9%) persons were tested for Ebola virus at CDC’s recommendation or by health department request; testing was not always performed for PUIs because alternative diagnoses were made or symptoms resolved. Of the 61 tested, 35 (57%) had traveled to an Ebola-affected country, 16 (26%) reported contact with an Ebola patient or their body fluids in the United States, and 10 (16%) had no Ebola risk factors but were tested at the request of the state or local health department (Table). Symptom history was available for 60 of the 61 persons tested; 56 (93%) had at least one sign or symptom consistent with Ebola. Specimens from 27 (44%) persons were tested at a state or local public health laboratory and confirmed at CDC, 15 (25%) were tested only at a public health laboratory and declared negative, and the remaining 19 (31%) were tested only at CDC.

Four persons were diagnosed in the United States with laboratory-confirmed Ebola; one died (7,8). Three were health care workers, two of whom provided intensive care to the first patient diagnosed in the United States. No secondary infections occurred among these four patients’ household or community contacts.

Among 33 recent travelers who tested negative for Ebola, alternative diagnoses were available for 13, the most common being malaria (n = 5) and viral illnesses (n = 4), including influenza. At least two persons who tested negative for Ebola died from other causes. Based on reports from health departments and health care providers, in several instances efforts to establish alternative diagnoses were reported to have been hampered or delayed because of infection control concerns. For example, laboratory tests to guide diagnosis or management (e.g., complete blood counts, liver function tests, serum chemistries, and malaria tests) were reportedly deferred in some cases until there were assurances of a negative Ebola virus test result. In other instances, radiologic studies, such as computed tomography and ultrasound scans, or evaluation for noninfectious conditions, such as severe hypertension and tachycardia, were reportedly delayed while a diagnosis of Ebola was under consideration.

On October 27, 2014, CDC implemented a risk-stratified active monitoring program for all travelers arriving from Ebola-affected countries to facilitate early detection of Ebola if these persons become ill (5). After arrival screening at designated U.S. ports of entry, health departments with jurisdiction at the travelers’ final destination perform daily active or direct active temperature and symptom monitoring§ for 21 days after the last possible Ebola exposure (6,9). Among the 650 inquiries described in this report, 107 (16%) occurred after active monitoring was instituted, and among these, 60 (56%) concerned persons who had traveled outside the United States, but only 17 (16%) had been to an Ebola-affected country in the preceding 21 days. Among these 17, a total of 14 were considered to be PUIs, because they had at least one sign or symptom consistent with Ebola. Upon evaluation of these 14 persons, nine were tested for Ebola and none were positive. Among the five who were not tested, an alternative diagnosis was made in three persons and symptoms resolved in two.

## Discussion

Since July 2014, CDC has provided enhanced consultation regarding potential Ebola cases to state and local health departments and health care providers throughout the United States, and has ensured that Ebola virus testing is widely available. As of December 3, 2014, a total of 44 state and local public health laboratories in 39 states and the District of Columbia are capable of conducting Ebola virus testing. This system of consultation and testing identified all four Ebola patients diagnosed in the United States. Each of these patients had Ebola-compatible clinical features and identifiable risk factors, highlighting the importance of a carefully obtained clinical and travel history. The combination of a high index of clinical suspicion by health care providers with expert consultation by state and local health departments and CDC has resulted in high sensitivity in detecting cases, which is of paramount importance to public health, especially for a disease as dangerous as Ebola.

The active monitoring program requires that all travelers from affected countries be monitored by local public health authorities for fever or other symptoms that might be early manifestations of Ebola for the duration of a 21-day incubation period. Symptomatic persons are referred and transported per protocols for Ebola (10,11) for clinical evaluation to a predetermined hospital that is prepared to assess and care for a PUI, thereby minimizing the possibility of secondary transmission and ensuring prompt evaluation and early initiation of treatment. From October 11, when entry screening began at U.S. airports, through November 15, a total of 2,263 travelers arriving from Guinea, Liberia, and Sierra Leone were screened, and none had symptoms during travel. One of these incoming travelers went on to develop Ebola; this person was asymptomatic at the time of entry screening, underscoring the value of post-arrival active monitoring as currently implemented.

The findings in this report are subject to at least two limitations. First, although this report describes all clinical inquiries received by CDC and accounts for all definitive Ebola virus tests conducted within the United States at public health or CDC laboratories, it does not represent all clinical inquiries received by health departments. Second, because clinical data were not systematically collected, information on certain variables might be incomplete.

A coordinated, national surveillance system facilitating the early detection of Ebola is an important defense against the possibility of importation and transmission within the United States and facilitates patients’ early access to medical care. CDC’s website provides up-to-date information on which travel areas might pose a risk (12). Clinicians should maintain a high index of suspicion and consult their local and state health departments and CDC when ill travelers from Ebola-affected countries are identified, although it is important to recognize that the likelihood of Ebola even among symptomatic travelers returning from these countries is very low. In the hospital setting, where policies and procedures should be in place to safeguard health care workers, consideration of Ebola should not delay diagnostic assessments, laboratory testing, and institution of appropriate care for other, more likely medical conditions.

What is already known on this topic***?***

The 2014 epidemic of Ebola virus disease (Ebola) is the largest Ebola epidemic ever known, with more than 6,000 deaths to date in Guinea, Liberia, and Sierra Leone. CDC offers consultation to state and local health officials and health care providers evaluating persons possibly at risk for Ebola. State-based, active monitoring of travelers arriving from Ebola-affected countries began on October 27, 2014.

What is added by this report?

During July 9–November 15, 2014, CDC responded to clinical inquiries regarding 650 persons in the United States. Sixty-one (9%) were tested for Ebola virus, and four were positive, including two travelers. Of the 17 persons who arrived in the United States from an Ebola-affected country after state-based active surveillance began, none had Ebola. Appropriate medical evaluation and treatment for other conditions were noted in some instances to have been delayed while a person was undergoing evaluation for Ebola.

What are the implications for public health practice?

State-based active monitoring facilitates the early detection of signs and symptoms among incoming travelers with known risk factors for Ebola. Health departments and health care workers should remain vigilant, but consideration of Ebola should not delay other indicated medical care.

## Figures and Tables

**FIGURE 1:**
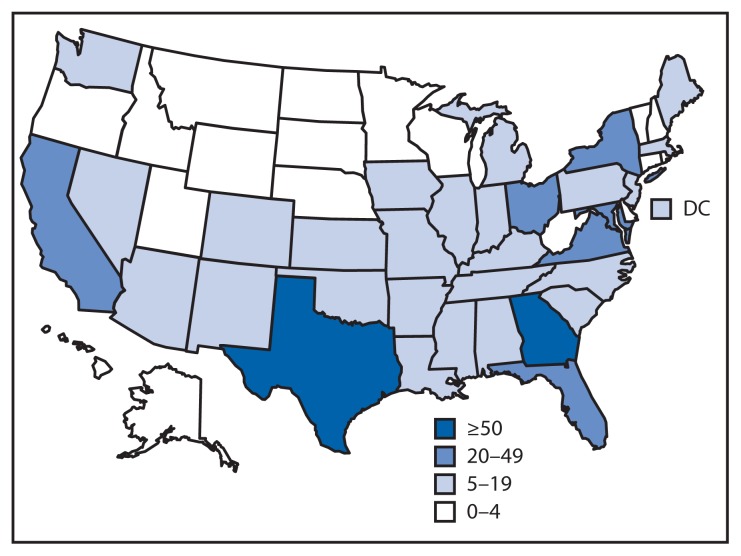
Number of clinical inquiries from health departments and health care providers regarding persons thought to be at risk for Ebola virus disease, by state — United States, July 9–November 15, 2014

**FIGURE 2:**
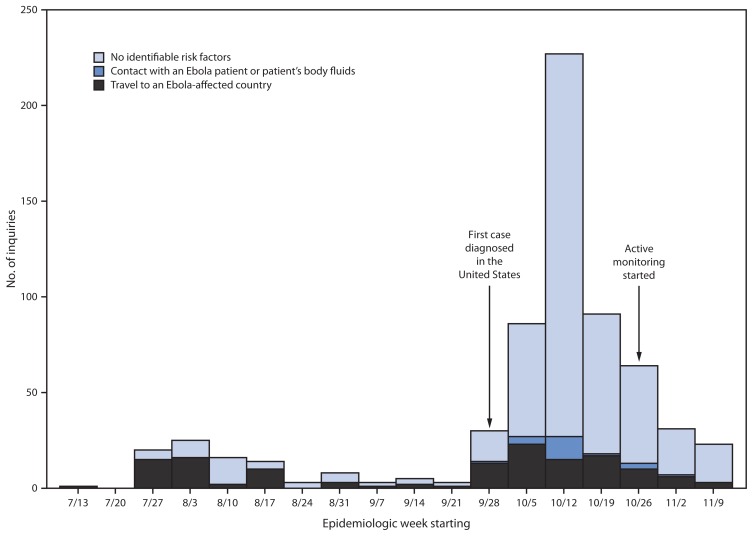
Number of clinical inquiries from health departments and health care providers regarding persons thought to be at risk for Ebola virus disease (Ebola), by epidemiologic risk factor^*^ and epidemiologic week — United States, July 9–November 15, 2014 ^*^ Epidemiologic risk factors include contact with an Ebola patient or patient’s body fluids or travel to an Ebola-affected country within 21 days of symptom onset. Countries with widespread Ebola virus transmission include Guinea, Liberia, and Sierra Leone. Those with localized transmission included Senegal during August 29–September 26 and Nigeria during July 23–September 30. Since October 18, Mali has had cases in urban settings.

**TABLE:** Clinical presentation and epidemiologic risk factors among persons under investigation for possible Ebola virus disease (Ebola)* who were tested for Ebola virus, by test result — United States, July 9–November 15, 2014

Characteristic	Test result			

	Positive (n = 4)		Negative (n = 57)*	
	
	No.	(%)	No.	(%)
**Clinical presentation**				
Subjective fever or core temperature ≥100.4°F (≥38.0°C)	4/4	(100)	40/56	(71)
Vomiting or diarrhea	2/4	(50)	25/56	(45)
At least one sign or symptom consistent with Ebola†	4/4	(100)	52/56	(93)
**Risk factor**				
Travel to an Ebola-affected country§	2/4	(50)	33/57	(58)
Contact with an Ebola patient or patient’s body fluids	2/4	(50)	14/57	(25)
No identifiable risk factors	0/4	(0)	10/57	(18)

* Symptom history was available for 56 of 57 persons who tested negative for Ebola virus.

† Signs and symptoms consistent with Ebola include elevated temperature (or subjective fever), severe headache, fatigue, muscle pain, vomiting, diarrhea, abdominal pain, or unexplained hemorrhage.

§ Countries with widespread Ebola virus transmission include Guinea, Liberia, and Sierra Leone. Those with localized transmission included Senegal during August 29–September 26 and Nigeria during July 23–September 30. Since October 18, Mali has had cases in urban settings.

## Footnotes

* Signs and symptoms consistent with Ebola include elevated temperature (or subjective fever), severe headache, fatigue, muscle pain, vomiting, diarrhea, abdominal pain, or unexplained hemorrhage. Many other conditions can cause combinations of these signs and symptoms.

† Epidemiologic risk factors include contact with an Ebola patient or patient’s body fluids or travel to an Ebola-affected country within 21 days of symptom onset. Countries with widespread Ebola virus transmission include Guinea, Liberia, and Sierra Leone. Those with localized transmission included Senegal during August 29–September 26 and Nigeria during July 23–September 30. Since October 18, Mali has had cases in urban settings.

§ Under active monitoring, state or local public health officials communicate daily with persons at risk for Ebola to check for the presence of fever or other signs or symptoms consistent with Ebola. Direct active monitoring means health officials conduct active monitoring through direct observation.
